# Fascinating Fasciclins: A Surprisingly Widespread Family of Proteins that Mediate Interactions between the Cell Exterior and the Cell Surface

**DOI:** 10.3390/ijms19061628

**Published:** 2018-05-31

**Authors:** Georg J. Seifert

**Affiliations:** Department of Applied Genetics and Cell Biology, University of Natural Resources and Life Science, Muthgasse 18, 1190 Vienna, Austria; georg.seifert@boku.ac.at; Tel.: +431-47654-94144

**Keywords:** arabinogalactan proteins, cellulose, pectin, matricellular proteins, SOS5, periostin, Mpb83

## Abstract

The Fasciclin 1 (FAS1) domain is an ancient structural motif in extracellular proteins present in all kingdoms of life and particularly abundant in plants. The FAS1 domain accommodates multiple interaction surfaces, enabling it to bind different ligands. The frequently observed tandem FAS1 arrangement might both positively and negatively regulate ligand binding. Additional protein domains and post-translational modifications are partially conserved between different evolutionary clades. Human FAS1 family members are associated with multiple aspects of health and disease. At the cellular level, mammalian FAS1 proteins are implicated in extracellular matrix structure, cell to extracellular matrix and cell to cell adhesion, paracrine signaling, intracellular trafficking and endocytosis. Mammalian FAS1 proteins bind to the integrin family of receptors and to protein and carbohydrate components of the extracellular matrix. FAS1 protein encoding plant genes exert effects on cellulosic and non-cellulosic cell wall structure and cellular signaling but to establish the modes of action for any plant FAS1 protein still requires biochemical experimentation. In fungi, eubacteria and archaea, the differential presence of FAS1 proteins in closely related organisms and isolated biochemical data suggest functions in pathogenicity and symbiosis. The inter-kingdom comparison of FAS1 proteins suggests that molecular mechanisms mediating interactions between cells and their environment may have evolved at the earliest known stages of evolution.

## 1. A Brief History of FAS1 Domain Proteins

The first FAS1 protein was identified in an insect model for central nervous system development, the grasshopper *Schistocerca americana.* In order to identify cell surface molecules potentially involved in the formation of axon bundles (fascicles), monoclonal antibodies (mAbs) recognizing cell surface antigens on specific fascicles were characterized. One of these antibodies recognized a 70 kDa glycoprotein named Fasciclin 1 (SaFas1 (Appendix A)) [1]. The genes coding for grasshopper SaFas1 and *Drosophila melanogaster* DmFas1 were cloned soon afterwards [2] and a homologous fruit fly gene called Midline fasciclin (*DmMfas*) was identified later [3]. In the fruit fly, a *DmFas1* knockout affected neuronal branching as well as synaptic function [4] and laser ablation of the grasshopper ortholog *SaFas1* led to disrupted cell adhesion of pioneer axons [5]. The crystal structure of DmFas1 provided the prototype for the structurally novel FAS1 domain [6]. In the meantime, molecular techniques and sequence comparison tools revealed the widespread occurrence of homologous proteins defined by the FAS1 domain (IPR000782; PF02469). The *Homo sapiens* genome encodes four FAS1 domain proteins named transforming growth factor-β induced protein (HsTgfbi), Periostin (HsPn), Stabilin-1 (HsStab1) and Stabilin-2 (HsStab2). The *HsTgfbi* gene (Appendix B) was identified in human adenocarcinoma cells as a transcript that was induced 20-fold by transforming growth factor-β [7]. Likewise, *HsPn*, was cloned based on its expression in an osteoblast cell line [8] and subsequently found to be enriched in the periosteum [9]. Finally, HsStab1 and HsStab2 were identified using two different antibodies binding to a subpopulation of endothelial cells and to the hyaluronic acid (HA) clearance receptor, respectively [10]. Due to their association with a multitude of clinical conditions, human and mammalian FAS1 proteins have been the focus of numerous detailed studies that are instructive for a general understanding of FAS1 domain proteins. Unexpectedly for proteins associated with cell adhesion, FAS1 domain proteins exist not only in animals but also in plants, fungi and prokaryotes. The first plant FAS1 protein was discovered using antibody interference in the alga *Volvox carteri* [11], a simple model for multi-cellularity consisting of just two cell types. When specific mAbs raised against a crude membrane preparation were added to volvox cultures they inhibited embryo development. The cognate protein was named algal cell adhesion molecule (CAM) based on its apparent role in the formation of intercellular contacts during early embryogenesis. The existence and physiological role of algal-CAM, which contains two FAS1 domains, raised the exciting possibility of a cell adhesion mechanism conserved between animals and plants. In higher plants FAS1 domain proteins were also identified by the biochemical and bioinformatic analysis of a group of highly *O*-glycosylated hydroxyproline-rich glycoproteins called arabinogalactan-proteins (AGPs) [12,13]. The bioinformatic investigation of the *Arabidopsis thaliana* genome revealed the existence of many fasciclin-like AGPs (FLAs) in plants [12,13,14]. At the same time a different investigation mapped one of several *Arabidopsis thaliana* salt overly sensitive (*sos*) mutations to the Salt overly sensitive 5 (*AtSos5*) gene encoding the AtFla4 protein [15]. In a mutant screen in a crop plant, the rice locus Microspore and tapetum regulator 1 (*OsMtr1*) was found to be required for male reproductive development and to encode a tandem FAS1 glycoprotein [16]. In fungi, FAS1 proteins have been identified in transcriptomics or proteomics studies in the Shiitake mushroom *Lentinula edodes* [17] and the rice pathogen *Magnaporthe oryzae* [18], while in the fission yeast *Schizosaccharomyces pombe* the FAS1 domain protein SpFsc1 was identified in a screen for autophagy related loci [19]. Apparently, FAS1 proteins already existed before the evolution of eukaryotes. The best-known eubacterial FAS1 proteins are Mpb70 and Mpb83, which were identified in *Mycobacterium bovis* culture filtrates [8,20,21,22,23,24]. Database queries reveal FAS1 proteins in both eubacteria and archaea, suggesting the inception of the domain preceded the existence of last universal common ancestor (LUCA) [25]. FAS1 proteins are often implicated in the interaction between the cell and the extracellular matrix (ECM). Considering the diversity of ECM architectures and compositions FAS1 domain proteins are surprisingly widespread between different kingdoms of uni- and multicellular life. However, despite their seemingly boundless presence throughout the tree of life, FAS1 proteins are not ubiquitous, especially in microbes whose genomes rapidly adapt to differing life styles. This suggests that FAS1 domain proteins are not essential for life per se but are suited for specialized cellular interactions that for some organisms are not required. I will next describe what is known about the structure of the FAS1 domain itself and discuss diverse additional structural features of FAS1 proteins in various kingdoms. This will be followed by a review of the biological roles of mammalian and plant FAS1 domain proteins, including the relationship of structure to function, which should help elucidate the mechanisms of FAS1 proteins in plant development.

## 2. The Structure of the Fasciclin 1 Domain

### 2.1. The Fasciclin 1 Domain

**Figure 1:**
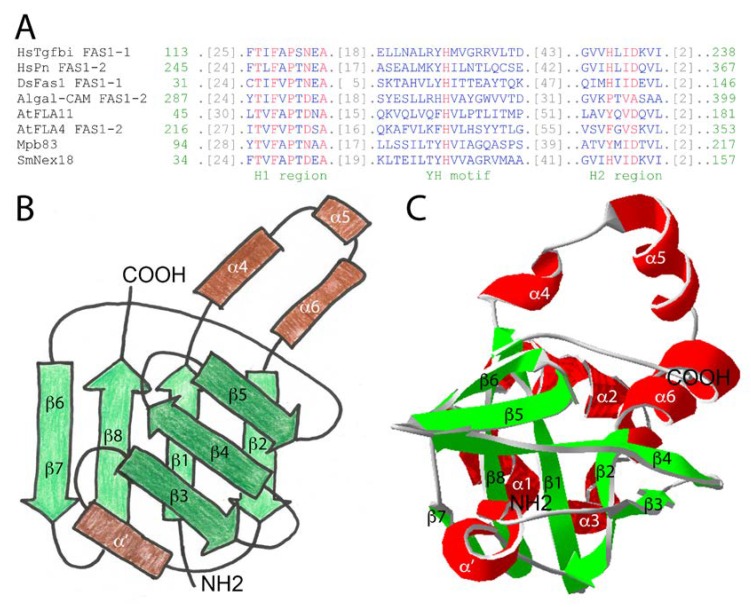
The FAS1 domain across kingdoms of life. (**A**) Delta-BLAST alignment of some FAS1 domains mentioned in this article. Note the conservation of the N and C-proximal H1 and the H2 region as well as the central YH motif. The sequences used by Delta-BLAST were HsTgfbi (NP_000349.1), HsPn (gi 93138709), DsFas1 FAS1-3 (1O70_A), Algal-CAM (gi 75282282), AtFLA11 (gi 116247778), AtFLA4 (gi 75206907), MbMpb83 (gi 614094354), and SmNex18 (gi 81635876). Red/blue indicates highly/not conserved residues; color bits threshold set to 2.5; (**B**) The general topology of the FAS1 domain features is reminiscent of the “thumbs-up” gesture. The secondary structure of HsTgfbi FAS1-1 is annotated omitting the three N-terminal helices for clarity; (**C**) The crystal structure of HsTgfbi FAS1-1 [33] in ribbon display.

The FAS1 domain extends to approximately 140 amino acids. Although sequence conservation between different FAS1 proteins can be quite low, there exist two more highly conserved sequence stretches of around 15 residues called H1 and H2 and a conserved central YH motif (Figure 1A). Therefore, to identify FAS1 domain proteins in sequence databases, domain enhanced lookup time accelerated BLAST (DELTA-BLAST) should be used [26]. Using X-ray crystallography and NMR spectroscopy, several studies have elucidated the structures of isolated FAS1 domains or of entire FAS1 proteins [27,28,29,30,31,32,33]. The FAS1 domain is globular and contains a central structural fold of two β-sheets oriented at an almost perpendicular angle, varyingly described as β-wedge or β-sandwich (Figure 1B). 

**Figure 2:**
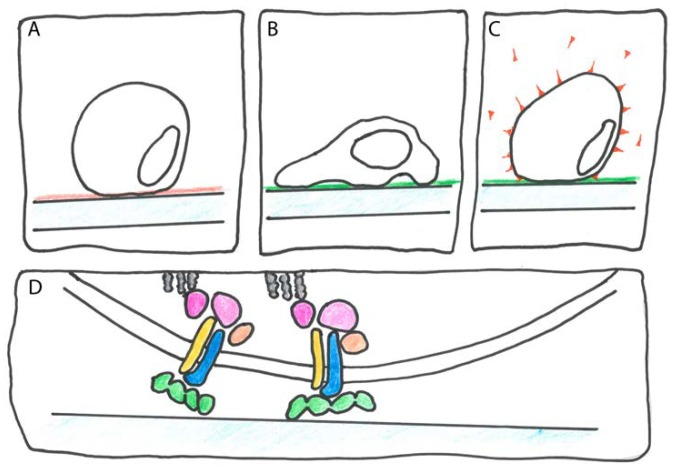
Cell adhesion assay. (**A**) On an uncoated or control-coated (red) plastic substrate, cell adhesion is inefficient; (**B**) cells adhere rapidly when plastic is coated with an adhesion protein such as Tgfbi or Pn (green); (**C**) to identify the receptor for the adhesion protein, integrin isotype specific antibodies (red) are co-incubated; (**D**) mammalian FAS1 proteins are thought to bind to different types of dimeric integrins (blue and yellow) that mediate mechanical contact between the cytoskeleton (grey) and the ECM as well as transduce intracellular signals using numerous associated proteins (pink and nude).

In the human HsTgfbi structure, the first β-sheet encompasses strands β1–β2–β8–β6/7 [33] (Figure 1C). The two inner strands are oriented parallel and the two outer ones antiparallel. The second β-sheet consists of β3–β4–β5. There are three α-helices at the N-terminus (not shown in Figure 1B), three more (α4 to α6) between β1 and β2 and a less highly conserved α-helix (α′) between β2 and β3. The FAS1 domain (Figure 1B, redrawn from [25]) is a member of the “β-grasp fold” superfamily [25] and may be imagined as a “thumbs-up” gesture of the right hand with the palm representing the first β-sheet (light green in Figure 1B), the bent index, middle and ring fingers symbolizing the second β-sheet (dark green in Figure 1B) and the thumb and pinkie resembling α4 to α6 (light brown in Figure 1B) and α′ (dark brown in Figure 1B), respectively. Among all FAS1 proteins, structure to function relations have been most intensely studied for HsTgfbi (reviewed in [34]). Therefore, the elucidation of the entire HsTgfbi crystal structure [33] could be seen as the “Rosetta stone” for a better mechanistic understanding of the many biological roles of FAS1 proteins in different organisms including plants. Several studies identified individual regions and amino acid residues on the four Tgfbi FAS1 domains that are critical for function. The main approach was to use in vitro cell adhesion as a functional assay (Figure 2).

Briefly, cells more efficiently adhere to surfaces coated with adhesion proteins such as Tgfbi than to control-coated surfaces (Figure 2A,B). That this adhesion is dependent on the family of ECM receptors called integrins can be tested by adding integrin antibodies that block the Tgfbi-stimulated cell adhesion (Figure 2C). To identify sites on Tgfbi that might mediate cell adhesion, peptides corresponding to conserved Tgfbi sequence motifs were added. For instance, the NKDIL and the EPDIM peptides that are part of the FAS1-2 and the FAS1-4 (see following section) domains, respectively, both interfered with Tgfbi stimulated cell adhesion. By contrast, the KADHH peptide in the corresponding region of FAS1-1 had no effect [35]. In a study of a different cell type expressing a different integrin, the NKDIL and EPDIM peptides did not interfere with adhesion but an 18-amino acid peptide that covered the YH motif did [36]. A HsPn specific mAb identified a corresponding integrin interaction region on FAS1-2 [37]. The cell adhesion assay is too crude to demonstrate *direct* binding between FAS1 proteins and integrin; however, in combination with the crystal structure of HsTgfbi it showed that different integrins interact with different surface regions of the FAS1 domains [33]. Although integrins are not known in plants, given the complex biological roles of some FAS1 proteins of plants, this insight should be valuable for the prediction of their molecular function.

### 2.2. Single and Tandem Fasciclin 1 Domains

**Figure 3:**
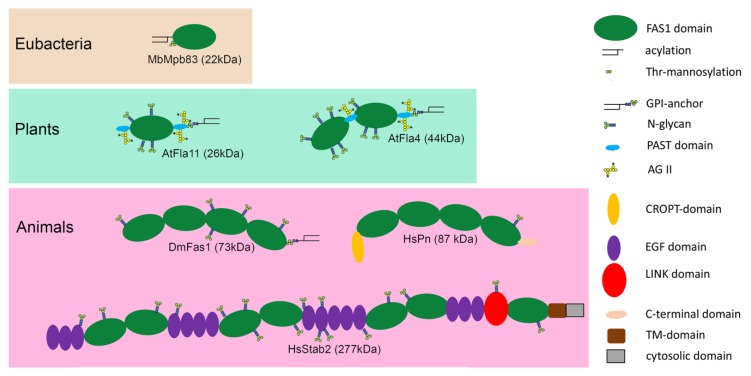
Domain organization and post translational modifications of six representative FAS1 proteins discussed in this article. Proteins are shown with their N-termini on the left. As opposed to the C-terminally attached GPI-anchors Mpb83 is acylated near its N-terminus. Note that the annotation of AG II glycosylation is tentative and that the annotation of *N*-glycans is based on bioinformatic predictions. Abbreviations used in this Figure, GPI: glycosylphosphoinositidyl; TM: transmembrane; AG II: arabinogalactan type II; CROPT: Cysteine-Rich domain of Pn and TGFBIp; EGF: epidermal growth factor; PAST: domain rich in Pro, Ala, Ser and Thr.

While prokaryotic representatives typically contain a single FAS1 domain, many eukaryotic family members contain two or more arranged in tandem (numbered FAS1-1, FAS1-2 etc. from N- to C-terminus). The two insect family members Fas1 and Mfas, and mammalian Tgfbi and Pn each contain four FAS1 domains, while there are seven in the two mammalian stabilins (Figure 3).

In plants, there exist both single FAS1 FLAs as well as family members with two domains in tandem. FAS1 tandem proteins also exist in fungi, where dual and five-fold tandems have been reported [18,19]. The tandem FAS1 domains structurally interact with each other, as was found in a structure of DmFas1 domains FAS1-3 an FAS1-4 [27] and of whole HsTgfbi [33]. It was suggested that the C-proximal domain in a tandem pair might bind to interaction partners such as integrins while the N-proximal domains might compete with this binding acting either as negative regulators or as safeguards against unwanted ligand binding [28]. A recent structure to function study of *AtFla4* in *Arabidopsis thaliana* showed that the C-proximal FAS1 domain was necessary and sufficient for genetic function [38]. By contrast, some studies showed cooperativity between all FAS1 domains [39]. To which degree the intramolecular interaction between neighboring FAS1 domains might affect the intermolecular interaction with other binding partners in vivo remains an open question.

### 2.3. Membrane Association

Most FAS1 domain proteins contain additional structural features and post-translational modifications some of which are shared between phylogenetically distinct family members. Practically all FAS1 proteins have an N-terminal secretion peptide; however, the final localization is determined by other signals. Mycobacterial Mpb83 is lipid-anchored to the plasma membrane by Cys acylation of the LAGC motif close to the N-terminus [40,41]. In plants, most FAS1 proteins are anchored to the outer sheet of the plasma membrane by a glycosylphosphoinositidyl (GPI) lipid anchor [14]. This modification is attached to proteins in the endoplasmic reticulum in exchange of a C-terminal signal peptide and exists in FAS1 proteins of plants, insects and fungi [17,18,42]. However, only one of the two *D. melanogaster* FAS1 proteins, DmFas1, is GPI-anchored [43]. The two mammalian stabilins and SpFsc1 from fission yeast are type 1 transmembrane proteins with cytosolic tails at their C-termini [10,19]. By contrast, Mpb70 from mycobacteria, mammalian Tgfbi and Pn, DmMfas and a group of FLAs are soluble proteins without any membrane anchoring motif [14,34]. Hence most FAS1 proteins are either bound to the plasma membrane or are soluble. In case of Mpb83 both surface anchored and soluble forms exist. Likewise, GPI-anchored proteins can be released from the plasma membrane by the action of phospholipases. The GPI anchor is probably needed for efficient transport of GPI-anchored proteins to the plasma membrane and potentially for their controlled release. In plants the GPI-anchor acts as a sorting signal for plasmodesmata [44]. Whether in non-animal eukaryotes such as plants and fungi that lack a cholesterol-rich plasma membrane, the GPI modification functions similar to animals to sequester proteins into membrane microdomains is under debate [45].

### 2.4. Glycosylation

Secreted proteins are often glycosylated. This is also the case for many FAS1 proteins, but not for all of them. For instance, although Mpb70 and Mpb83 share 75% sequence identity, but the latter is *O*-glycosylated close to its N-terminus with three threonine-linked mannose residues [46]. Another example is the *N*-linked glycosylation of four animal FAS1 proteins, mammalian Tgfbi and Pn and insect Fas1 and Mfas that are similar in size and all have four FAS1 domains, however, they carry zero, one, five and eight *N*-glycans, respectively. Likewise, in plant FAS1 proteins, *N*-glycosylation of FAS1 domains ranges from two to eight *N*-glycans [14,16]. A distinguishing feature of FAS1 proteins in plants is the presence of regions rich in proline, alanine, serine and threonine (PAST). Especially sequences containing the repetitive pattern (P-A/S/T)_n_, whose Pro residues are post-translationally converted to Hyp and galactosylated are typical for AGPs [47]. Indeed, the first FLAs were identified in precipitates obtained with β-glucosyl Yariv reagent [12,13], which binds to β(1→3) galactose chains present on many AGPs [48,49]. However, the peptide patterns for *O*-glycosylation are not as precisely defined as the N-X-T/S *N*-glycosylation motif [50], and biochemical identification of *O*-glycosylation on defined proteins is not as straightforward as the molecular weight shift caused by enzymatic removal of *N*-glycans. Hence one should be aware of potential discrepancies as to which FAS1 proteins are indeed FLAs, e.g., Algal-CAM contains an extensive PAST-rich N-terminal region and so does OsMtr1, and after removing *N*-glycans the apparent molecular weights of both proteins were larger than expected for the apoprotein [11,16]. Therefore, both proteins might well be members of the FLA family. By contrast, other FAS1 proteins such as the wheat locus named *TaFLA9* lack any PAST-rich regions [51] and might be wrongly annotated as FLAs. However, the AtFla4 protein is decorated with AGP-like glycan epitopes despite the absence of canonical (P-A/S/T)_n_ repeats [38]. Clearly, the simple presence or absence of PAST-rich regions is an inaccurate predictor for AGP-like *O*-glycosylation. The *O*-glycans of FLAs and other AGPs are known as type II arabinogalactans (AG II). They consist of Hyp-linked galactose that is the basis of a (1–3) β-linked galactan backbone with (1–6) β-linked galactose branches and kinks. AG II contains terminal modifications, mainly by l-arabinofuranose, l-rhamnose, l-fucose, d-glucuronic acid, 4-methyl-d-glucuronic acid and d-xylose [52,53,54]. Although pharmacological and genetic interference with AG II function has revealed many biological roles for these carbohydrates [55,56], models of how AGPs function remain sparse. An AGP of low abundance was shown to form a covalent link with both hemicellulose and pectin [57]. In fractionated cell walls, AGP-associated carbohydrate epitopes are abundant in pectic and hemicellulosic fractions and some are even present in the cellulose fraction [58]. Moreover, AGPs are self-adhesive in a calcium-dependent fashion [59], and can bind to pectin via carboxyl–calcium ion bridges [60]. Such results indirectly suggest that AG II-modified proteins FLAs might at least partially be linked to cell wall polysaccharides via their *O*-glycans. 

### 2.5. Additional Domains

For human FAS1 proteins, *O*-glycosylation has not been reported [43]; however, they contain additional domains that expand their spectrum of molecular interactions. The N-terminal region of mature HsTgfbi, HsPn and DsMfas folds into a structure called Cysteine-Rich domain of Pn and TGFBIp (CROPT) [33], that mediates the interaction with various forms of collagen, fibronectin and the heterophilic binding between HsPn and HsTgfbi (reviewed in [43,61]). The human stabilins contain three dual FAS1 domain tandems and one isolated FAS1 domain, that each are separated by a varying number of Epidermal Growth Factor (EGF) repeats and an X-link domain. These domains are thought to be involved in phosphatidylserine and hyaluronic acid binding, respectively, but are not present in FAS1 proteins other than mammalian stabilins [62,63]. The C-terminal domain (CTD) of HsTgfbi contains the RGD motif that, although crucial for integrin binding of many adhesion proteins, is not required for the adhesion stimulating properties of HsTgfbi [64,65]. However, the RGD motif might modulate binding of HsTgfbi to specific integrins [39]. The CTD of HsPn, displays a high degree of intrinsic disorder and is both differentially spliced [66] and proteolytically regulated [67]. Interestingly, compared to full-length forms, HsPn forms lacking the CTD show relatively enhanced binding to ECM proteins [68]. The extreme C-terminus is highly basic and is thought to bind to heparin. Differential splicing and proteolysis might switch between forms of HsPn that more readily binds to HS and forms that more strongly interact with matrix proteins [61,67]. Compared to vertebrates, differential splicing is less frequent in plants; however, a great degree of regulatory fine-tuning might be provided by differential gene expression of the large number of plant genes encoding FAS1 proteins.

### 2.6. Groups of Fasciclin 1 Proteins in Plants

Compared to other organisms, plants contain the greatest number of FAS1 protein encoding genes. The majority of them are annotated as FLAs. The numbers of FLA encoding loci vary considerably between different plant genomes such as cotton (**17** and **19** loci in *Gossypium barbadense* and *G. hirsutum*, respectively [69,70]), *Eucalyptus grandis* (**18** [71]), *Arabidopsis thaliana* (**21** [14]), *Cannabis sativa* (**23** [72]), *Brassica rapa* (**33** [73]), wheat *Triticum aestivum* (**34** [51]), rice *Oryza sativa* (**27** [74]) and cottonwood *Populus trichocarpa* (**35** [75]). Apparently, diversity in this gene family varies even between closely related species (e.g., between the two *Gossypium* species or between *Arabidopsis thaliana* and *Brassica rapa*). An important step to understand the diversity of this complex gene family was sub-classification according to the presence of one or two FAS1 domains, their sequence similarity, the presence of GPI-anchors and the organization of PAST-regions [14]. Inclusion of FAS1 domain proteins that are not yet annotated as AGPs, such as OsMtr1 and Algal-CAM, might slightly enlarge the FLA family. The present FLA sub-classification has four groups: group A members are GPI-anchored and contain a single FAS1 domain flanked by two PAST-regions, group B are not GPI-anchored and sport a tandem of two FAS1 domains separated by a PAST-region. Group C contains GPI-anchored, single and tandem FAS1 proteins, with the single FAS1 domains phylogenetically closest to the N-proximal FAS1 domains of that group. Moreover, group C has PAST regions inserted close to their C-termini and between FAS1 domains. Lastly, group D contains FLAs that are not closely related to any other group and might represent separate branches of diversification.

## 3. Biological Aspects of FAS1 Domain Proteins across the Tree of Life

### Human and Mammalian Fasciclin 1 Proteins

FAS1 proteins have been the subject of experimentation in a variety of species; however, by far the most work has been published about the four human representatives [reviewed in [34,43,76,77,78,79,80]]. Particularly *HsPn*, *HsTgfbi* and *HsStab2* have been implicated in a plethora of medically relevant conditions ranging from normal development [76,81] to wound healing [77], bone regeneration [82,83] and cancer [67]. A Medline search shows 624, 1229, 105 and 718 entries for Tgfbi, Pn, Stab1 and Stab2, respectively (2 March 2018, Title/Abstract, synonyms see Appendix B). A detailed medical discussion is beyond the scope of this article; however, selected highlights are presented to provide a critical viewpoint on the molecular function of FAS1 domain proteins in plants. Missense mutations in the *HsTgfbi* locus are known to cause different types of corneal dystrophy [84]. In many cases of this disease, mutated HsTgfbi forms aggregates in the corneal stroma. This is due to the thermal and pH instability of the protein and to its high abundance in the cornea [85]; however, the condition might not help us to understand HsTgfbi function. A complete *Tgfbi* gene knockout in mice caused neither corneal abnormalities nor any other major defects [86], suggesting compensation by other ECM molecules. Although *Pn* knock-out mice showed that this locus is essential for normal osteogenesis and dental development [87,88], the two closely-related *Pn* and *Tgfbi* loci function identically in many ways [43]. The most intriguing common property of mammalian FAS1 proteins is their interaction with the integrin family of ECM receptors (Table 1). Hetero-dimeric integrins are expressed in a cell-type-specific fashion and bind to many different ECM proteins such as fibronectin, laminin and fibrinogen. The cell adhesion assay described above (Figure 2) suggests that FAS1 domains might bind to integrins; however, to establish the mode of binding, alternative assays such as co-immunoprecipitation of recombinant proteins and Foerster resonance energy transfer are required (Table 1). 

**Figure 4:**
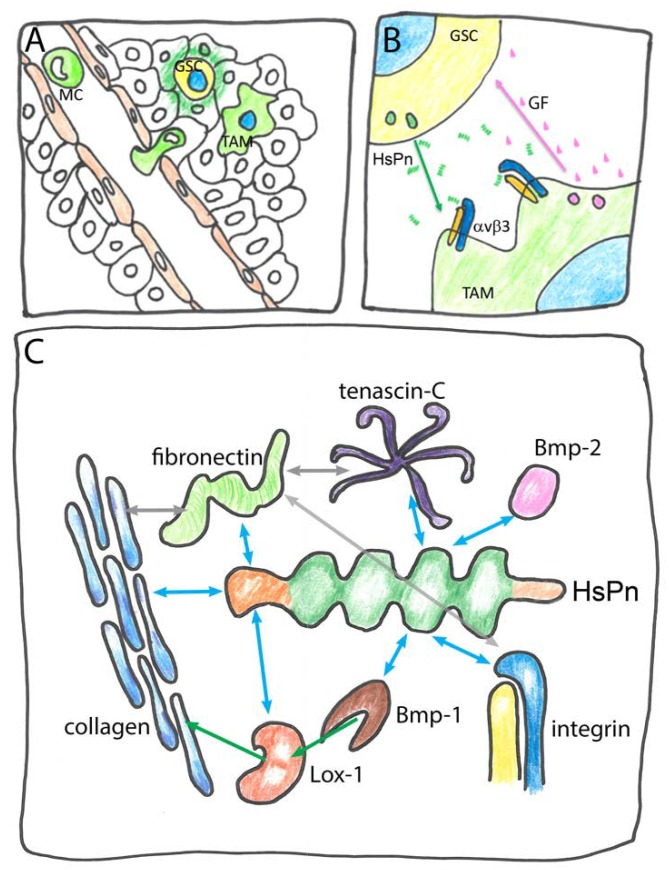
Multiple roles of HsPn: (**A**) Circulatory monocytes (MC) are attracted by glioblastoma stem cells (GSC) that secrete HsPn (dark green) to invade the site of the primary tumor and differentiate into tumor associated macrophages (TAM); (**B**) GSC-secreted Pn via integrin αvβ3 signaling triggers the release of growth factors (GF) that establish a metastatic niche; (**C**) molecular interactions of Pn in the ECM. Blue double arrows indicate binding of HsPn. Grey double arrows indicate binding of other components. Green arrows indicate activating reactions: the metalloprotease Bone morphogenetic protein Bmp-1 proteolytically activates Lysine oxidase 1 (Lox-1) in turn enhancing collagen crosslinking.

The integrin complexes are hubs of mechano-chemical signaling and interact with the cytoskeleton and with numerous signaling pathways including focal adhesion kinase (FAK), mitogen activated protein kinase (MAPK), Target of rapamycin (mTOR), and β-catenin that influence cell division and migration, differentiation and programmed cell death [76]. FAS1 proteins not only bind and influence integrins as a stationary component of the ECM but also act as mobile paracrine factors. An example for the latter is the role of HsPn in glioblastoma. In this invasive brain cancer, glioblastoma stem cells (GSCs) secrete HsPn that acts as chemoattractant for circulatory monocytes (MCs)—precursors of macrophages (Figure 4A).

MCs are induced to invade the site of the primary tumor and to differentiate into tumor associated macrophages (TAMs) by HsPn binding to MC-expressed integrin. In turn, TAMs secrete growth factors that further stimulate cell division in GSCs generating a metastatic niche [105] (Figure 4B). A similar model for Pn function has previously been proposed for other malignancies such as cholonangiocarcinoma and renal carcinoma [93,104]. Both HsPn and HsTgfbi directly bind to several ECM components and modulate the biosynthesis of the ECM proteins (Figure 4C). Owing to its versatile binding properties, mammalian Tgfbi was previously known as collagen-associated protein containing the RGD sequence (RGD-CAP) [111], while HsPn is known to bind to collagen and fibronectin [61]. The latter interaction affects the correct secretion and deposition of these fibrous proteins [112] and additional interactions facilitate the intramolecular crosslinking of collagen in the ECM. Via its FAS1-1 and FAS1-3 domain Pn binds to Bone morphogenetic protein-1 (Bmp-1), a metalloprotease that proteolytically activates lysine oxidase-1 (Lox-1), which, in turn, enhances collagen crosslinking [113,114]. The peptide growth factor Bone morphogenetic protein-2 (Bmp-2) that is not related to Bmp-1 also binds to Pn albeit at a different site [113]. The physiological role of this interaction remains unknown. Another matrix protein interacting with Pn is tenascin-C that forms a remarkable six-armed structure named hexabrachion and it was suggested that the link between tenascin-C and collagen formed by Pn might adapt the ECM to mechanical stress [68]. While some of the ligands of HsPn and HsTgfbi bind to the CROPT domain or the C-terminal domain, the interaction with integrin occurs via FAS1 domains. In overview, the two secreted FAS1 proteins HsTgfbi and HsPn exemplify how FAS1 domains accommodate a variety of protein–protein interactions enabling a single protein to act both in ECM integration and in intercellular signaling. By contrast, the membrane anchored stabilins mostly act as receptors for self ligands and as cell–cell interaction signals. One of their biological functions is in the removal of unwanted material from extracellular spaces and circulation by professional scavenger cells. Despite their similarity at the domain and sequence level, HsStab1 and HsStab2 are associated with distinct ligands and processes. HsStab1 acts as an intracellular sorting receptor as well as an endocytotic cell surface receptor for ligands such as acetylated low-density lipoprotein (acLDL) [115], secreted protein acidic and rich in cysteine (SPARC) [116,117], placental lactogen (PL) [118] and a chitinase-like protein called SI-CLP [119,120]. The FAS1-7 domain of HsStab1 binds to PL and SI-CLP while acLDL and SPARC interact with domains other than FAS1. HsStab1 can guide its various ligands on the secretory route from the Golgi into the lysosomes after endocytosis and through transcytosis (reviewed in [79]). HsStab2 binds ligands such as acLDL and advanced glycation products as well as various glycosaminoglycans such as HA, chondroitin sulfate and dermatan sulfate as well as the anticoagulant heparin and is required for the physiological clearance of these materials from circulation [121]. The polysaccharides bind to the X-link domain on Stab2 [122]. Both HsStab1 and HsStab2 have also been implicated in the phagocytosis of unwanted cells [123]. With their EGF domains, the stabilins recognize the “eat-me!” signal phosphatidylserine (PS) present at the surface of apoptotic cells and aged erythrocytes. HsStab2 utilizes its FAS1 domain in this process to bind to and cooperate with αvβ5 integrin [107]. The interaction between HsStab2 and PS is important for the fusion of uninucleate myoblasts during myofiber formation with PS functioning as a “fuse-me!” signal [124,125]. Finally, the interaction of HsStab2 FAS1 domains with integrins mediated both homotypic and heterotypic cell to cell adhesion [110,126]. In summary, each of the four human FAS1 proteins fulfils a variety of different biological functions. While secreted HsTgfbi and HsPn act in cell–ECM adhesion, ECM structuring and intercellular signaling, the membrane-anchored HsStab1 and HsStab2 proteins act in endocytosis, intracellular trafficking and cell to cell recognition.

## 4. Biological Functions of Plant Fasciclin 1 Proteins

The varying gene numbers for FLAs in different plant species shows that gene duplication and gene loss are common. On the one hand, this suggests that the large repertoire of FAS1 proteins might be fine-tuned at the level of differential gene expression analogous to differential mRNA splicing in vertebrates. On the other hand, gene redundancy makes the investigation of FLA function more cumbersome. However, the non-redundant *AtFla1*, *AtFla3*, *AtFla4, OsMtr1* and the pair of *AtFla11* and -*12* provide a glimpse of the role of FAS1 proteins in plants.

### 4.1. The AtFla4 AtFei Pathway

**Figure 5:**
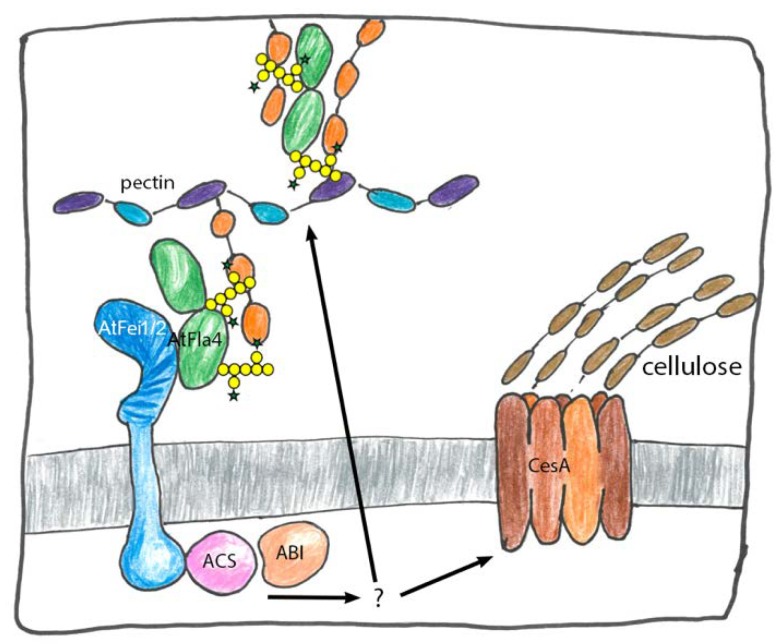
Hypothetical roles of AtFla4 in cell wall structure and signaling. AtFla4 might interact with the pectic network with covalent or non-covalent interactions of its glycans (yellow discs and green stars: *O*-glycans which are yet to be precisely defined, *N*-glycans are omitted). It might mechanically link pectin with AtFei1 and AtFei2 receptor kinases and the plasma membrane. The signals transduced by the receptor kinases might involve ACC synthase (ACS) and Abcisic Acid Insensitive 1 (ABI1). How signaling connects the kinases with cellulose synthase (CesA) and pectin biosyn thesis is unclear.

A mutant screen in *Arabidopsis thaliana* that discovered several key regulators of salt stress response [127] also identified a mutant that carried a missense mutation in the *AtFla4* locus [15] often called *atsos5* and here referred to as *atfla4* for consistency. In *atfla4* root elongation was almost completely stalled after transfer to growth media containing moderate levels of NaCl and the mutant root developed a characteristic radially swollen tip. Likewise, elevated levels of sucrose induced this phenotype in *atfla4* [128], which is reminiscent of many conditional mutants defective in cell wall polysaccharide biosynthesis [129]. Although conditional root swelling is the most dramatic aspect of the *atfla4* phenotype, the mutant’s abnormally thickened hypocotyls and inflorescence stems, its fat roots under salt-free conditions, larger leaves, shorter siliques and its abnormal seed coat mucilage indicate that *AtFla4* acts non-conditionally throughout the plant [15,128,130,131,132,133,134]. Cell walls in *atfla4* roots were shown to lack the pectin-rich middle lamella that is essential for intercellular adhesion, suggesting a potential function of *AtFla4* in pectin biosynthesis or structure [15]. However, double knockouts in the two similar leucine-rich receptor-like kinases (LRR-RLKs) *AtFei1* and *AtFei2* were not only phenotypically identical to *atfla4* but also non-additively interacted with it [128]. This suggested that *AtFla4* and the two *AtFei* loci act in a linear genetic pathway. The *atfei1 atfei2* double mutants were hypersensitive to cellulose biosynthesis inhibition and showed a decrease in crystalline cellulose production under restrictive conditions. As these phenotypic features were reminiscent of cellulose biosynthetic mutants it was proposed that the Fei-RLKs and by association AtFla4, might function in the biosynthesis of this important polymer [128]. The phenotype of both *atfla4* and *atfei1 atfei2* was modulated by variety of growth regulators. Firstly, the *atfei1 atfei2* root phenotype was suppressed by inhibition of the biosynthesis and oxidation of ACC but not of the perception and signaling of its product ethylene [128]. This suggested that, in line with *AtFla4*, the *AtFei* loci might negatively regulate a novel ethylene-independent ACC signaling pathway that is activated by cell wall defects [135]. Both AtFei proteins interacted with ACC synthase in yeast two hybrid assays but did not phosphorylate them in vitro [128]. Secondly, abscisic acid (ABA) signaling acted synergistically with *AtFla4* to regulate root elongation and abiotic stress response as the *atfla4* phenotype was suppressed by exogenous ABA and by ABA-oversensitive mutants and a *atfla4*-like phenotype was induced by ABA inhibition [130]. Interestingly, *AtFla4* did not interact with two NADPH respiratory burst oxidases that are required for stress responses triggered by cellulose synthase inhibition [136,137]. Finally, both the *atfei1 atfei2* double and the *atfla4* single mutants non-additively interacted with the IAA-alanine resistant 4 (*AtIar4*) locus [138]. These studies implicate the *AtFla4 AtFei* pathway with a variety of intracellular signaling pathways; however, they do not provide a model for the relation between the cell wall and AtFla4. Insights into this problem were stimulated by the characterization of the seed coat mucilage phenotype of *atfla4* and *atfei2*. During seed maturation, pectic mucilage polymers are secreted into a pocket between the plasma membrane and the primary cell wall. Upon hydration of the mature seed the mucilage polymers rapidly swell and rupture the primary walls. However, the mucilage normally adheres to the ruptured primary cell wall [139]. There exist numerous mutants affected in aspects of seed coat mucilage biosynthesis and maturation. They are instructive models for cell wall biosynthesis throughout the plant [140]. Because in *atfla4* and *atfei2* mutants and in the cellulose synthase mutant *atcesA5* seed coat mucilage adhesion was found to be defective it was initially suggested that *AtFla4* and *AtFei2* might act in cellulose biosynthesis [131,132]. However, the subsequent comparative analysis of *atfla4*, *atfei2, atcesA5* and other mucilage adherence mutants showed distinct roles of *AtFla4* and *AtFei2* on the one hand and *AtCesA5* on the other hand [133,134]. Rather than influencing cellulose or hemicellulose structure, *AtFla4* acted on the pectin network, which was clearly distinct from the genetic actions of *AtCesA5* and of the xylan biosynthetic locus Mucilage-modified5 (*AtMum5*) [133,141,142]. In contrast to mutants in the latter genes, *atfla4* non-additively interacted with two pectin-related loci. Firstly, Mucilage-modified2 (*AtMum2*) encodes a β-galactosidase acting on galactan side chains of pectic rhamnogalacturonan type I (RG I) and its loss of function prevents the extrusion of mucilage upon hydration [143]. The extrusion defect of *atmum2* was suppressed in the *atfla4 atmum2* double mutant [133]. Secondly, Flying saucer 1 (*AtFly1*) encodes a transmembrane E3 ubiquitin ligase thought to regulate the degree of pectin methyl esterification. Loss of *AtFly1* function led to ectopic adhesion between primary cell walls and mucilage [144] and *atfla4* suppressed this phenotype [133,134]. A possible explanation for these observations is that AtFla4 might physically interact with galactan side chains on rhamnogalacturonan type I (RGI) pectin structures that are normally remodeled by the β-galactosidase encoded by *AtMum2*, to control timely mucilage maturation and adherence. This hypothesis is consistent with the localization of *AtFla4* in the mucilage pocket during seed coat development [133,134]. How does AtFei2 fit into this scenario, and can the characterization of the seed coat mucilage phenotype be extrapolated to the whole plant? In the case of seed coat mucilage, AtFei2 might attach AtFla4 to the plasma membrane and thereby contribute to the architecture of the network—an entirely structural explanation. However, it cannot be ruled out that the interaction between the hypothetical AtFla4-pectin matrix and AtFei2 might also convey signals to the cell interior [133,134]. While AtFla4 localized to the developing mucilage pocket and the adjacent plasma membrane of seed coat epidermal cells, AtFla10 and AtFla17 but not AtFla4 were identified in a proteome of mature mucilage [145]. Whatever the biological function of these FLAs in mature mucilage, due to the *atfla4* single mutant phenotype they apparently are not functionally equivalent to AtFla4. It is unclear whether the mode of action of *AtFla4* in seed coat mucilage is identical to its mechanism throughout the plant; however, the overlapping genetic role with the *AtFei* genes in both tissues suggests a common mechanism. The AtFla4 protein might interact with various cellular components. It was hypothesized that AtFla4 might bind to the AtFei RLKs [128,146]. The localization of the majority of yellow fluorescent protein-tagged AtFla4 at the plasma membrane is compatible with this possibility [38]. However, AtFla4 was also released into the apoplast, where it might interact with cell wall carbohydrates [38,133]. The interaction of AtFla4 with cell wall polysaccharides remains to be investigated; however, both covalent and non-covalent interactions between pectic polymers and AG II glycan have previously been demonstrated (see above). In fact, it was shown that protein *O*-galactosylation is required for the function of AtFla4. Several galactosyl transferases (GALTs) mediate the initial glycosylation of hydroxyl proline residues of AGPs. Two of them called AtGalt2 and AtGalt5 together are responsible for the biosynthesis of the bulk of AGPs and their mutation caused a root and seed phenotype identical to *atfla4* [147]. Intriguingly, the phenotypes of *atgalt2 atgalt5 atfla4 atfei1 atfei2* quintuple and the *atfla4 atfei1 atfei2* triple mutants were identical [146]. It was hypothesized that AtFla4 might physically interact with the Fei RLKs via its *O*-glycans. This hypothesis was seemingly contradicted by a structure to function study where the deletion of potential *O*-glycosylation sites on AtFla4 did not interfere with genetic complementation of *atfla4* [38]. However, the AtFla4 level in the transgenic plants might have been higher than normal or cryptic *O*-glycosylation sites might still have been present. How AtFla4 and AtFei interact and if *O*-linked glycosylation plays a direct or indirect role for AtFla4 and AtFei function remains to be tested. Taken together, *AtFla4* is a unique genetic paradigm for FAS1 domain proteins in plants. A speculative model (Figure 5) combined from previous studies proposes that after its release from the plasma membrane and via its *O*-glycan AtFla4 possibly interacts with pectic polymers and contributes to biophysical properties such as swelling and interpolymer connectivity. 

In parallel AtFla4 might also interact with AtFei1 and AtFei2 via its FAS1 domain to link pectin with AtFei1 and -2 and indirectly to ACC synthase. The access of the C-terminal Fas1 domain to the AtFei receptor domain might be modulated by the interaction to the cell wall. Under standard conditions relatively firm binding of AtFla4 to AtFei might suppress ACS. Under cell wall stress conditions binding of AtFla4 to AtFei might be reduced and the ACS suppression by AtFei might be relaxed leading to ACC production and decreased cellulose production and other responses. However, any postulated physical interactions of *AtFla4* remain to be demonstrated.

### 4.2. A Conserved Function of AtFla11 and AtFla12 in Secondary Cell Walls

One of the functionally and economically most interesting aspects of some FLAs is their role for secondary cell wall formation and structure. Secondary cell walls are by far the most abundant re-growing fiber and energy feedstock on this planet and its major constituents—cellulose, xylans and lignin—are among the most abundant biopolymers. Their sustainable and socially compatible production is thought crucial in the global transition to a carbon neutral economy. *AtFla11* and *AtFla12* (group A; [14]) and their orthologs (Appendix C) from diverse fiber crops and model plants were noted in *Arabidopsis thaliana* for their expression during the secondary thickening of stems [148], their co-expression with secondary cell–wall–specific cellulose synthase loci [149] and their expression in sclerenchyma cells [150]. A correlative and causative association of *AtFla11* and *AtFla12* orthologs with secondary cell wall biosynthesis has been observed in many plant species. The *Zinnia elegans ZeFla11* gene was found to be expressed in trans-differentiating cell cultures and in a specific subset of secondary cell wall forming vessel cells and adjacent parenchyma cells [151]. *AtFla11* and -*12* orthologs from poplar, eucalyptus and willow were highly expressed in tension wood (TW), a reaction wood forming at the upper side of branches and stems growing sideways. TW consists of almost pure cellulose deposited in the gelatinous secondary cell wall layer (G-layer) in elongated G-fiber cells and shows a characteristically low-cellulose microfibril angle (MFA, with respect to the growth axis), which entails superior tensile strength [152,153,154]. The three *AtFla11/12* eucalyptus orthologs *EgFla1*, -*2* and -*3* were highly expressed in stems correlating with secondary cell wall formation [71]. Also in flax and hemp *AtFla11/12* orthologs were highly expressed in fiber forming tissues [72,155]. A cotton *AtFla12* ortholog was expressed at a higher level and for a longer time period in *Gossypum barbadense* compared to *G. hirsutum* correlating with greater fiber strength and length of the former [69]. Hence, *AtFla11* orthologs might be natural genetic determinants for fiber crops. So, throughout the higher plants, *AtFla11/12* and their orthologs correlate with secondary cell wall formation, specifically the deposition of cellulose microfibrils aligned along the axis of mechanical stress. What is the role of FLAs in this process?

In *Arabidopsis thaliana* there exists a genetic redundancy between *AtFla11* and -*12*; however, the *atfla11 atfla12* double mutant was affected in the mechanical properties of its secondary cell–wall–rich stems [156]. In *atfla11 atfla12* tensile strength and stiffness were reduced and MFA was increased. Moreover, the chemical composition and cell wall structure showed a reduction of galactose, arabinose and cellulose and a concomitant increase in lignin content. The authors speculated that the FLAs via their FAS1 domains might form a heteromeric higher-order network strengthening the interaction between cellulose microfibrils [156]. The most highly expressed hybrid aspen locus called *PtFLA6* was silenced with antisense RNA. This also reduced the transcript level of many other *PtFla* genes [157,158]. Although no apparent morphological phenotype was observed, the *PtFla6* knockdown resulted in reduced flexural strength and stiffness in stems of transgenic plants. Moreover, the content of AGPs, cellulose and lignin was reduced alongside a reduction of transcript levels for numerous genes involved in cellulose and lignin formation [157]. When TW formation was stimulated in the *PtFla6* knockdown plant, the relative frequency of abnormal G-fibers increased, but no other changes were observed [158]. Taken together, the complex response to *PtFla6* interference is more compatible with a regulatory role than it is with a direct structural role in the cellulose layer. In *Eucalyptus grandis, EgFla1*, -*2* and -*3* were experimentally overexpressed in cambial sectors or in whole tobacco plants [71]. Despite their sequence similarity, each transgene exerted a specific effect on phenotypic parameters such as MFA, cell wall thickness, cell wall composition and xylem cell type specification. This meant that structurally even very similar *AtFla11/12* orthologs exerted different effects in the same cellular context [71]. The function of *AtFla11/12* orthologs has also been investigated during cotton fiber formation. In this system, trichome cells are initiated at the outer integument of the ovule during anthesis (2 d before to 5 d after anthesis; dpa). Then the trichomes undergo a period of rapid cell elongation (3–20 dpa), which is followed by secondary wall formation (16–40 dpa) and maturation (40–60 dpa). Hence, each cotton fiber is a single trichome cell made up of almost pure cellulose [159]. Its formation is the result of three distinct developmental processes: (1) specification and initiation, (2) expansion and (3) cell wall thickening and maturation. Two *AtFla11/12* orthologs in the cotton species *Gossypium hirsutum* named *GhAgp4* (Appendix D) and *GhFla1* were suppressed using RNAi [159,160]. *GhFla1* was specifically expressed in elongating fibers with peak expression at 10 dpa [70] and indeed, fibers of RNAi lines were much shorter than controls [159]. However, *GhFla1* RNAi suppression also resulted in delayed and reduced trichome initiation and, moreover, resulted in decreased RNA levels of other fiber-expressed FLAs and of numerous genes implicated with cell wall biosynthesis. The complex alterations at the transcript level correlated with alterations in cell wall composition including decreased cellulose formation. By contrast, the overexpression of *GhFla1* resulted in increased initiation and elongation of cotton fibers as well as upregulation of other FLAs and cell wall biosynthetic loci and in an increased cellulose content [159]. These data confirmed and extended a previous study where the *GhAGP4* locus was silenced by RNAi. Like the GhFla1 silencing, this reduced fiber initiation, elongation and quality, and affected the level of transcripts related to cell wall biosynthesis [160]. The observations made in cotton are difficult to reconcile with a biomechanical role of *GhFla1* and *GhAgp4* as an adhesive between secondary cell wall cellulose microfibrils because not only secondary cell walls were affected by the interference but also the fundamentally different processes of initiation and elongation that happen before. The apparent involvement of these genes in all three phases of fiber formation and their control of mRNA levels for an entire battery of other genes rather suggests that this class of FAS1 proteins fulfils a role in developmental control comparable to a peptide hormone such as the proteoglycan xylogen [161].

**Figure 6:**
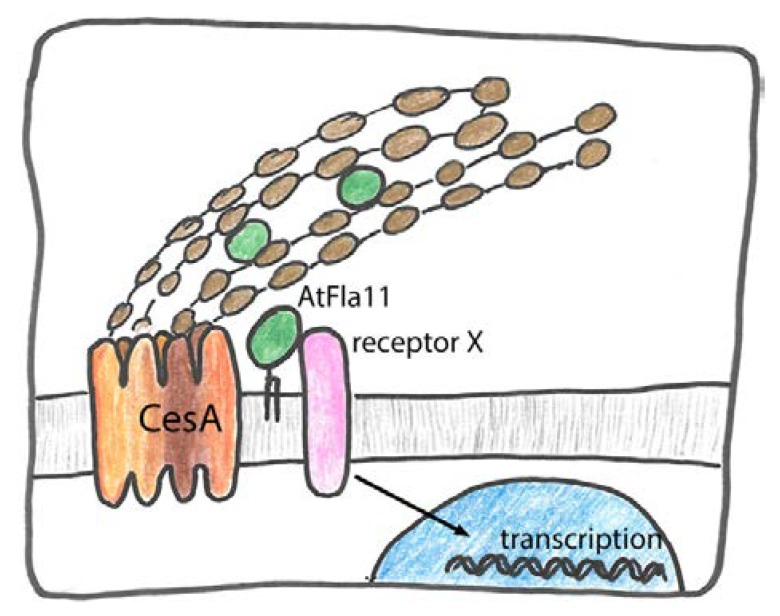
Putative role of *AtFla11*, *AtFla12* and their orthologs in secondary cell wall formation. Firstly they might act as secreted structural proteins in secondary cell walls binding to cellulose or other glycans. Secondly, they might be part of outside–in signaling, in connection with an unknown receptor that leads to complex changes of gene expression. Thirdly, they might bind to cellulose synthase (CesA) and directly influence or stabilize its action. Finally, they might act as adaptors between CesA and receptor kinases to modulate CesA phosphorylation.

What do we know about possible molecular interactors and subcellular localization of *AtFla11/AtFla12* orthologs? Intriguingly, cotton orthologs of *AtFla7* and *AtFla11* were co-precipitated with active secondary cellulose synthase *GhCesA8* that was effectively extracted by inclusion of cellulase in the extraction buffer [162]. This supports the hypothesis that group A FLAs physically interact with the cellulose synthase complex. This hypothesis offers an attractive explanation for co-expression between group A FLAs and secondary cell wall formation related genes and for changes in MFA and mechanical properties in group A *FLA* loss of function genotypes. If one assumes that nascent cellulose fibers in combination with cellulose synthase are both bound by FLAs, this model could also explain the localization of PtFLA6 in secondary cell walls [157,158]. However, this model offers no simple explanation for the observation that the transcriptional program of secondary cell wall formation as well as cellular differentiation was heavily influenced by the experimental manipulation of FLAs [157], or for the many effects of FLA overexpression. This problem might be solved with a role of FLAs in signaling. So far, there has been one report that suggested that a RLK might physically interact with FLAs. A pull-down experiment using the *Arabidopsis thaliana* sucrose-induced receptor kinase (AtSirk1) as bait identified AtFla8 and AtFla9 among the 16 interaction partners. Interestingly, among several potential substrates of AtSirk1, there was the primary cell wall specific cellulose synthase catalytic subunit 3 (AtCesA3) [163]. At present, there is no genetic evidence linking AtFla8 and AtFla9 with primary cell wall cellulose synthesis; however, the evidence for their physical interaction with an RLK that acts on cellulose synthase offers an interesting alternative mechanism for how FLAs might influence cell wall biosynthesis. Using their potential to interact with multiple binding partners they might combine receptor kinases with substrates such as cellulose synthase. Taken together, several modes of action can be envisaged for *AtFla11/12* and its orthologs (Figure 6).

Firstly, AtFla11/12 might be directly secreted to the cell wall and bind to polymers and cross-link them. Secondly these FAS1 proteins might act as extracellular signals or be part of a receptor complex to influence transcriptional events downstream. Thirdly, they might augment cellulose deposition by acting as a physical part of the cellulose synthase machinery. Fourth, by interacting with both cellulose synthase and receptor kinase they might facilitate or modulate post-translational regulation of cellulose biosynthesis (Figure 6).

### 4.3. A Cascade of FLAs Acts in Male Gametophyte Development

**Figure 7:**
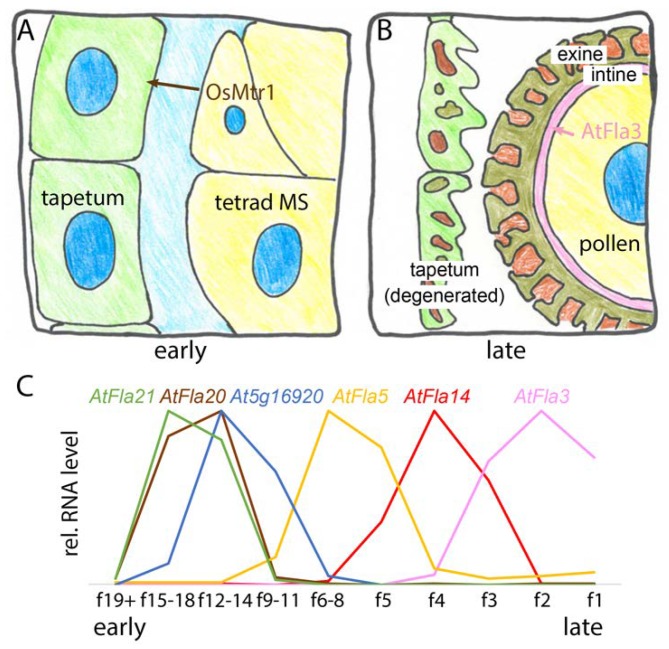
FAS1 proteins act sequentially in male gametophyte development: (**A**) *OsMtr1* acts in early stage of tapetum development. It is specifically expressed and secreted in microspore cells (MS) and influences the development of tapetum cells; (**B**) *AtFla3* acts at the late stage of the formation of the cellulose- and pectin-rich intine layer of pollen cell walls; (**C**) RNA-Seq data of *Arabidopsis thaliana* at different floral stages [164,165] show that six FAS1 genes are specifically expressed in anthers at different stages of floral development. f1 indicates the first open flower. The expression patterns of AtFla20 that might be the ortholog of OsMtr1 and of AtFla3 are consistent with roles in early and late pollen development, respectively.

The FAS1 proteins encoded by the *AtFla3* and the *OsMtr1* genes, respectively, are required for male gametophyte development; however, the mRNA pattern in *Arabidopsis thaliana* suggests that several FLAs might sequentially act in this process (Figure 7).

No functional pollen was found in the rice microspore and tapetum regulator 1 (*osmtr1*) mutant [16]. *OsMtr1* expression was specifically detected in early microspore development during meiosis and tetrad formation and during the release of young microspores (Figure 7A). However, despite *OsMtr1* never being expressed in the tapetum, a nutritive maternal tissue that surrounds microspores, the *osmtr1* mutation dramatically affected tapetum development. This suggested that *OsMtr1* might act non-cell autonomously. The *osmtr1* mutant tapetum failed to undergo programmed cell death, which normally supports pollen wall formation in developing microspores. Tetrads formed normally in *osmtr1* yet, mutant microspores showed defective development as soon as they were released possibly due to the lack of cell wall material contributed by the adjacent tapetum. The localization of OsMtr1 in the microspores and the extracellular space between them and the tapetum suggested that OsMtr1 might act as a secreted signal to coordinate microspore development with tapetum degeneration (Figure 7A). Molecular interactors such as a hypothetical *OsMtr1* receptor on tapetum cells remain to be identified [16]. The expression of *AtFla3* in pollen during anthesis and pollen germination combined with the high level of expression in anthers is unique among all FLAs and *AtFla3* function was investigated using RNAi and overexpression [166]. The *AtFla3* RNAi lines were partially male-sterile due to a reduced ability to form functional microspores. Histological analysis showed that *AtFla3* RNAi affected the formation of intine, the innermost layer of the pollen wall, which consists mainly of cellulose and pectin. The earliest time point at which intine abnormalities were observed was the late uninucleate stage, suggesting that *AtFla3* might act in the formation of intine in microspore development. Abnormal *AtFla3* RNAi pollen showed defective cellulose staining, suggesting that *AtFlaA3* was required for cellulose deposition in the intine layer. More detailed analyses are required to test whether any other cell wall polymers are affected and if full loss of function of the *AtFla3* locus causes a more complete defect of pollen function. During *Arabidopsis thaliana* floral development at least six FAS1 domain loci are specifically expressed in anthers during separate developmental stages [164,165]. Like its closest rice homologue *OsMtr1, AtFla20* is expressed at an early stage, where it overlaps with *AtFLA21* and *At5g16920*—an FAS1 protein not annotated as FLA (Figure 7C). The mRNA levels of *AtFla14* and *AtFla5* sequentially peak at intermediate stages and *AtFla3* is expressed immediately before and during flower opening (anthesis) in mature and germinating pollen, as previously reported [166]. How most of these FAS1 domain proteins are involved in normal pollen wall formation remains to be investigated. Nonetheless, their expression pattern during pollen formation appears to reflect the complex development and composition of the pollen wall.

### 4.4. Plant FAS1 Proteins Have Many Potential Functions

Several other FLAs are known to perform at least partially non-redundant genetic roles. Firstly, the loss of function mutation of *AtFla1* caused a phenotype in the growth regulator induced formation of shoots and roots from the callus [167]. Two alleles in two different wild-type backgrounds were isolated and the wild-type background on its own not only had a dramatic influence on callus derived organ formation but it essentially inverted the genetic effect of *AtFla1* on the process. By contrast, the negative effect of the *AtFla1* locus on root length and lateral root number was constant between the two tested wild-type backgrounds. It is presently unclear how *AtFla1* might influence organ formation but initial promoter studies showed expression of *AtFla1* early during the formation of organized tissue from callus. The protein might therefore be required for pattern formation [167]. Secondly, in a gene-editing study in the non-model plant *Brassica carinata, BcFla1*, a group A FLA that is more similar to *AtFla13* than to *AtFla11,* was implicated in root hair growth, where tip-focused deposition of primary cell wall materials occurs [168]. In a third example, in maize the expression level of group A *ZmFla* genes was inversely correlated with seed abortion. Consistently, two different mutations in the group A *AtFla9* locus that showed abnormally low and high *AtFla9* RNA levels displayed increased and decreased seed abortion, respectively [169]. Lastly, a comparative study of 30 different wheat (*Triticum aestivum*) varieties found an association between the expression of a FAS1 protein and grain milling properties [170]. According to this report, the locus *TaFla8* [51] coding for a FAS1 domain that does not contain any PAST-rich domains, is specifically expressed in grain. Interestingly, its level was relatively low in varieties displaying beneficial milling properties compared to varieties scoring poorly with respect to milling efficiency. Hence, human selection of wheat varieties with improved milling properties might have inadvertently reduced the basal expression level of *TaFla8*, which might normally strengthen cell walls. 

## 5. Fungal FAS1 Proteins

In a screen for genes specifically expressed in fruiting bodies of the Shiitake mushroom *Lentinula edodes* the *LeFlp1* locus coding for a GPI-anchored single FAS1 domain protein was identified and its specific expression during fruiting body formation suggested a developmental role [17]. In another study the *MoFlp1* locus of the rice blast fungus *Magnaporthe oryzae* was shown to encode a GPI-anchored vacuolar tandem FAS1 protein [18]. Its disruption caused defects in the formation of conidia, which, according to the authors, hinted at a potential role of *MoFlp1* in autophagy. Interestingly, the fission yeast *Schizosaccharomyces pombe* FAS1 domain protein SpFsc1 was identified in a genetic screen for autophagy-related loci [19]. SpFsc1 consists of five FAS1 domains in tandem and a transmembrane domain. The protein was detected in the vacuolar membrane and was shown to specifically act in a late step in autophagy, the fusion between the phagosomes with the vacuolar membrane. The mechanistic details including the role of the FAS1 domains in this process remain to be elucidated. In summary, studies on FAS1 domain proteins in fungi are sparse; however, they provide a stimulating variation of the hypothetical modes of action of this protein family.

## 6. Bacterial FAS1 Proteins

The most thoroughly studied eubacterial FAS1 proteins are the homologues Mpb70 and Mpb83 from *Mycobacterium* (reviewed in [171]). In various genomes of obligate pathogenic, opportunistic and non-pathogenic mycobacteria [172], both non-pathogenic and pathogenic species contained *Mpb70/Mpb83* homologues, while the genomes of opportunistic mycobacteria did not. This suggests a relation of *Mpb70/Mpb83* to mycobacterial lifestyle and pathogenesis. While Mpb83/Mpb70 was hypothesized to undergo “homophilic” interactions with mammalian Pn thereby interfering with cell adhesion in bones [171] this idea is not compatible with the secreted nature of Pn and is derived from cell aggregation data [8,173], which could have alternative explanations. Moreover, no intermolecular interactions between Fas1 domains have been reported. However, both host cell to ECM adhesion and host cell to mycobacterium adhesion might be modulated by binding of Mpb70/Mpb83 to molecules presented at the host cell surface. Indeed, Mpb83 was demonstrated to bind to Toll-like receptors 1 and -2 (HsTlr1 and -2), two LRR-RLKs present on human monocytes [41]. The binding of recombinant Mpb83 to HsTlr2 triggered mitogen activated protein kinase (MAPK) signaling and as a consequence induced the production of matrix metalloprotease-9 (MMP-9) and cytokines [174]. The direct interaction between Tlr2 and Mpb83, as shown by surface plasmon resonance [41], is an intriguing finding, given the presence of more than 200 LRR-RLKs in plant genomes [175]. A role of bacterial FAS1 proteins in the interaction between bacteria and their eukaryotic hosts is also observed in symbiotic communities. In the first example, the FAS1 protein SmNex18 (locus SMa1077) of the nitrogen-fixing legume symbiont *Sinorhizobium meliloti* was shown to be expressed specifically during nodulation, the initial stage of symbiosis between bacterium and plant [176,177]. Secondly, a FAS1 protein was found in *Nostonoc* cyanobacteria that form symbiotic bacterial–fungal communities called lichen. *Nostonoc punctiforme* isolates living in lichen highly expressed an unnamed FAS1 protein (locus ACC81089) that was only a minor compound in a closely related free living strain [178]. Moreover, FAS1 proteins featured prominently in the metaproteome of phyllospheric (i.e., plant leaf dwelling) bacteria [179] but were absent from rhizospheric (root-dwelling) bacteria [180]. Likewise, while present in many different genera of archaea, the FAS1 domain is not necessarily ubiquitous within the same genus. To give an example, among the 13 completely sequenced *Methanobacterium* isolates, only *M. paludis* isolated from peatland contained FAS1 domain protein sequences [181]. On the one hand, such circumstantial observations support the hypothesis that FAS1 proteins are important for the interaction between prokaryotic colonizers and eukaryotic hosts; on the other hand, they also show that FAS1 proteins are not essential.

## 7. Concluding Remarks

Can we define a common denominator for the function of FAS1 proteins? The characterization of HsTgfbi structure and function demonstrates the versatility of the FAS1 domain in binding multiple ligands, which might be a conserved feature. The frequent assumption that FAS1 proteins are involved in “cell adhesion,” however, is more context-dependent. It is derived from insightful experiments performed with animal cell cultures; however, it might be misleading for “hard-shelled” cells such as bacteria, fungi and plants that usually adhere to their ECM as a result of turgor pressure and build up adhesive materials such as pectin. By contrast, the designation of Tgfbi and Pn as matricellular proteins, defined as non-structural ECM-components that interact with cell surface receptors as mediators between the cell and its microenvironment [77,182], seems a more productive concept for approaching the common function of FAS1 proteins in all kingdoms of life including plants. The crucial challenge for future work on plant FAS1 proteins will be the identification of molecular interactors both of the FAS1 domain and of their abundant but enigmatic glycan modifications.

## Figures and Tables

**Table 1:** Mammalian Fas1 proteins interact with integrin receptors.

Integrin Type	TGFBI	PN	STAB-2
α1β1	[64] I,A		
α2β1	[89] C,F		
α3β1	[35] A,I,P [90] A,I [91] A,I		
α4	[92] I		
α5	[92] I		
α5β1		[93] A,I,G	
α6β4	[94] A,I	[95] I	
α7β1	[96] A,I		
αvβ3	[97] A,I,P [98] A,I [99] C,F	[100] I,A [101] I [88] I [102] A,D [103] G [104] I [105] I	
αvβ5	[106] A,I,P [36] A,I,P	[100] I,A [101] I [102] A,D [104] I	[107] C,T
β3		[108] G,A	
αMβ2	[65] A,C,I	[109] A,I	[110] I,G

Assay type: G: genetic interference (siRNA, transfection, mutants & epistasis analysis etc.); A: cell adhesion (coating of plastic surfaces); I: immuno-interference (e.g., anti-integrin antibodies inhibit PN mediated responses); P: peptide interference (peptide of Fas1 domain interferes with integrin dependent binding); C: Co-IP (endogenous or added recombinant Fas1 protein); F: immunofluorescence co-localization; D: DNA-aptamer inhibition; T: FRET.

## Appendix A

Since many genes and proteins from different organisms are discussed in this review, I adopted universal nomenclature rules according to a highly popular textbook [183]. Gene symbols are written in italics, with the first letter in uppercase indicating a wild-type allele and in lowercase indicating a mutant allele. Proteins are written in non-italics. If the text relates to genes or proteins from a certain species, a two-letter prefix (Hs for *Homo sapiens,* At for *Arabidopsis thaliana* etc.) is used.

## Appendix B

Because many human proteins were independently discovered several times, and maybe also for asserting the status of discovery, there is an abundance of synonyms. Tgfbi is also known as β-ig H3, βIG, BIGH3, kerato-epithelin, RGD-CAP and MP78/70. Pn is synonymous with Osteoblast specific factor-2 (Osf-2). Stab1 has also been named Common lymphatic endothelial and vascular endothelial receptor-1 (Clever-1) and FEEL-1 is an acronym for fasciclin, EGF-like, laminin-type EGF-like, and link domain-containing scavenger receptor-1. Frequent Stab2 synonyms are lymphatic vessel endothelial hyaluronan receptor-1 (LYVE-1) and hyaluronic acid receptor (HARE).

## Appendix C

The term “*AtFla11/12* ortholog” is here used to designate FLAs from other plant species that are more similar in protein sequence to *AtFla11* or *AtFla12* than to any other *Arabidopsis thaliana* protein.

## Appendix D

The nomenclature in the cotton literature is somewhat confusing. Some FLAs are named AGPs and GhFla1 refers to two entirely different protein sequences. The GhFla1 that was mentioned by Li et al. (2010) [160] being most similar to *AtFla9/6/13* and the *GhFla1* silenced by Huang et al. (2013) [159] is an ortholog of *AtFla11/12*. The *GhAgp4* locus (gb EF470295) silenced by Li (2010) [160] is identical to the sequence annotated as *GhFla12*.
